# Supplementary figures and images for: Isolation, cryo-laser scanning confocal microscope imaging and cryo-FIB milling of mouse glutamatergic synaptosomes
Source: PLoS One. 2022 Aug 12;17(8):e0271799. doi: 10.1371/journal.pone.0271799 (PMC9374259; doi:10.1371/journal.pone.0271799)

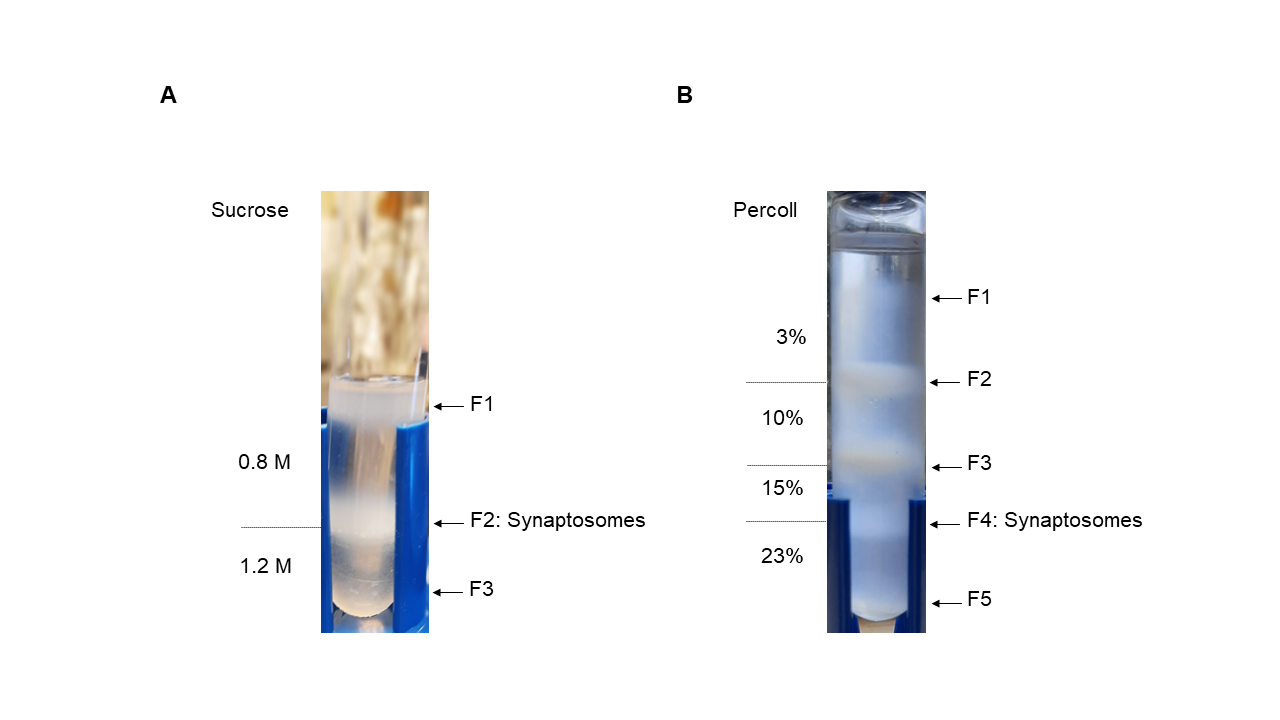

Supplement: S1 Fig — (A) Typical appearance of centrifuge tubes after sucrose density gradient centrifugation. The fraction F2 at the interface between 0.8 and 1.2 M sucrose corresponds to synaptosomes. A similar result is observed for Ficoll density gradient centrifugation with the synaptosome fraction (F2) lying between 8 and 14% Ficoll. In case of sucrose and Ficoll density gradient centrifugation, fractions F1 and F3 represent myelin & membranes and extrasynaptosomal mitochondria, respectively [33,36]. (B) Typical appearance after Percoll density gradient centrifugation. Fraction F4 at the interface between 15 and 23% Percoll corresponds to synaptosomes. Fractions F1, F2, F3 and F5 contain membranes, myelin & membranes, synaptosomes along with membrane vesicles and extrasynaptosomal mitochondria, respectively [38]. (TIF) [file pone.0271799.s001.tif]
