# Supplementary material for: Isolation, cryo-laser scanning confocal microscope imaging and cryo-FIB milling of mouse glutamatergic synaptosomes
Source: PLoS One. 2022 Aug 12;17(8):e0271799. doi: 10.1371/journal.pone.0271799 (PMC9374259; doi:10.1371/journal.pone.0271799)
Supplement: S1 File — (PDF) [file pone.0271799.s002.pdf]

Isolation, cryo-laser scanning confocal microscope imaging and cryo-FIB milling of mouse glutamatergic synaptosomes

Prerana Gogoi[1], Momoko Shiozaki[2], Eric Gouaux[1,3]

[1]Vollum Institute, Oregon Health and Science University, Portland, OR, USA;

[2]Howard Hughes Medical Institute, Janelia Research Campus, Ashburn, VA, USA;

[3]Howard Hughes Medical Institute, Oregon Health and Science University, Portland, OR, USA

dx.doi.org/10.17504/protocols.io.kxygxz5mkv8j/v1

Eric Gouaux

ABSTRACT

Ionotropic glutamate receptors (iGluRs) at postsynaptic terminals mediate the majority of fast excitatory neurotransmission in response to release of glutamate from the presynaptic terminal. Obtaining structural information on the molecular organization of iGluRs in their native environment, along with other signaling and scaffolding proteins in the postsynaptic density (PSD), and associated proteins on the presynaptic terminal, would enhance understanding of the molecular basis for excitatory synaptic transmission in normal and in disease states. Cryo-electron tomography (ET) studies of synaptosomes is one attractive vehicle by which to study iGluR-containing excitatory synapses. Here we describe a workflow for the preparation of glutamatergic synaptosomes for cryo-ET studies. We describe the utilization of fluorescent markers for the facile detection of the pre and postsynaptic terminals of glutamatergic synaptosomes using cryo-laser scanning confocal microscope (cryo-LSM). We further provide the details for preparation of lamellae, between ~100 to 200 nm thick, of glutamatergic synaptosomes using cryo-focused ion-beam (FIB) milling. We monitor the lamella preparation using a scanning electron microscope (SEM) and following lamella production, we identify regions for subsequent cryo-ET studies by confocal fluorescent imaging, exploiting the pre and postsynaptic fluorophores.

DOI

dx.doi.org/10.17504/protocols.io.kxygxz5mkv8j/v1

PROTOCOL CITATION

Prerana Gogoi, Momoko Shiozaki, Eric Gouaux . Isolation, cryo-laser scanning confocal microscope imaging and cryo-FIB milling of mouse glutamatergic synaptosomes. **protocols.io** https://dx.doi.org/10.17504/protocols.io.kxygxz5mkv8j/v1

FUNDERS ACKNOWLEDGEMENT

NINDS
Grant ID: 2R01NS038631

Howard Hughes Medical Institute
Grant ID: N/A

KEYWORDS

Ionotropic glutamate receptors, Postsynaptic density, density gradient centrifugation, lamella, fluorescence imaging, synapse

LICENSE

This is an open access protocol distributed under the terms of the Creative Commons Attribution License, which permits unrestricted use, distribution, and reproduction in any medium, provided the original author and source are credited

CREATED

Apr 28, 2022

LAST MODIFIED

May 02, 2022

PROTOCOL INTEGER ID

61656

MATERIALS TEXT

Reagents

- Adult mouse brain: All vGLUT1-mVenus knock-in mice (both male and female) were euthanized under Institutional Animal Care and Use Committee (IACUC) protocols, consistent with the recommendations of the Panel on Euthanasia of the American Veterinary Medical Association (AVMA) and carried out only by members of Dr. Gouaux's lab approved on the IACUC protocol TR01_IP00000905.
- Sucrose (Sigma, cat. no.84097)
- Ficoll 400 (Sigma, cat. no. F2637)
- Percoll (GE Healthcare, cat. no. 17-0891-02)
- HEPES (Fisher Bioreagents, cat. no. BP310-1)
- Leupeptin (Sigma, cat. no. L0649)
- Aprotinin (Sigma, cat. no. A1153)
- Pepstatin A (Sigma, cat. no. P4265)
- Gold fiducial beads, 10 nm (Ted-Pella, Inc., 90010-5)
- 50%/50% ethane and propane mixture
- Digitonin (Apollo Scientific, cat. no. 11024-24-1)

Equipment

- Dissection tools
- 18-G needle (18G x 1 ½) (BD Precision Glide, Cat. no. 305196)
- Dispensing needle, 14G (C-U Innovations)
- Glass Teflon dounce homogenizer

- 38.5 mL, open-top thinwall ultra-clear tube, 25 x 89mm (Beckman Coulter, cat. no. 344058)
- Nalgene™ high-speed polycarbonate round bottom centrifuge tubes (Thermo scientific, cat. no. 3117-0120)
- 10.4 mL, polycarbonate bottle with cap assembly, 16 x 76mm (Beckman Coulter, cat. no. 355603)
- SS-34 fixed angle rotor (Sorvall)
- Type 75 Ti fixed angle rotor (Beckman Coulter)
- Type 45 Ti fixed angle rotor (Beckman Coulter)
- SW27 swinging bucket rotor (Beckman Coulter)
- RC5C refrigerated floor centrifuge (Sorvall)
- Optima L-80 XP ultracentrifuge (Beckman Coulter)
- Optima MAX-XP ultracentrifuge (Beckman Coulter)
- Zeiss LSM 980 with Airyscan 2 (Zeiss)
- Cryo-correlative microscopy stage (Linkam, cat. no. CMS196V)
- Aquilos 2 Cryo-FIB (Thermo Scientific)
- R 2/1 Holey Carbon-coated 200-mesh gold grids (Quantifoil Micro Tools GmbH, cat. no. Q51340)
- Blotting paper (Ted pella, Inc., cat. no. 47000)
- Steriflip-GP sterile centrifuge tube top filter unit (Millipore, cat. no. SCGP00525)
- 70 mL, Polycarbonate bottle assembly, 38 x 102 mm (Beckman Coulter, cat. no. 355622)
- Autogrid or C-Clip ring (Thermo Scientific, cat. no. 1036173)
- C-Clip (Thermo Scientific, cat. no. 1036171)
- AutoGrid container (Thermo Scientific, cat. no. 1084591)
- Thermobarrel extruder (Lipex, Transferra Nanosciences Inc.)
- Cytiva Whatman nucleopore hydrophilic membrane, 1 μm (Fisher, cat. no. 18400175)
- Fluorescence-detection size-exclusion chromatography (FSEC) [56].

Reagent setup

The volume of reagents used in this protocol is optimized for synaptosome preparation from three adult whole mouse brains (each brain ~0.5 gm)

Homogenization buffer (HB)

Freshly prepare 50 mL homogenization buffer (HB) by mixing 10 mM HEPES pH 7.4 and 0.32 M sucrose. Add protease inhibitors to final concentrations of 0.8 μM aprotinin, 2 μg/mL leupeptin and 2 μM pepstatin A to the HB. Filter the HB using a Steriflip-GP sterile centrifuge tube top filter unit (0.22 μm) and store in ice until use.

Density gradient buffers

Sucrose gradient buffer: Prepare 10 mL each of 0.8 and 1.2 M sucrose in HB.
Ficoll gradient buffer: Prepare 10 mL each of 8 and 14% (w/v) Ficoll in HB. Percoll gradient buffer: Prepare 5 mL each of 3, 10, 15 and 23% (v/v) Percoll in HB.

Tris-buffered saline (TBS)

Mix 20 mM Tris HCl pH 8.0 and 150 mM NaCl; filter the buffer using 0.2-μm filter and store at room temperature (RT; 25 ºC).

Solubilization buffer

Freshly prepare solubilization buffer by mixing 20 mM Tris HCl pH 8.0, 150 mM NaCl and 2% (w/v) digitonin. Allow the digitonin to dissolve into the buffer solution by placing it on a nutator overnight at 4 ºC. The digitonin containing buffer will appear cloudy and can be used directly without filtration at this stage. Immediately before use, add aprotinin, leupeptin and pepstatin A to final concentrations of 0.8 μM, 2 μg/mL and 2 μM, respectively.

FSEC buffer
Mix 20 mM Tris HCl pH 8, 150 mM NaCl and 0.075% (w/v) digitonin. Allow digitonin to dissolve into the buffer solution by placing on a magnetic stirrer overnight at 4 ºC. Centrifuge at 9,700*g* for 20 min at 4 ºC to remove residual digitonin followed by filtering the buffer using a 0.2-µm filter. Store the buffer at 4 °C for up to one week and filter each time before use.

10 nm gold fiducial
Centrifuge 500 µL of the 10 nm gold fiducial solution at 21,130*g* for 30 min in a benchtop centrifuge. Remove the supernatant without disturbing the soft pellet of the gold beads using a pipette. Add 500 µL phosphate-buffered saline (PBS) pH 7.4 buffer to wash the gold beads and subject it to another round of centrifugation at 21,130*g* for 30 min followed by removing the supernatant using a pipette. Prepare the final stock of the gold fiducial beads at an optical density (OD) of 15-10 by adding PBS pH 7.4 to make the final volume of 20 µL.

Equipment setup

Centrifuge
The success of the density gradient separation of synaptosomes is critically dependent on the centrifuge, rotors and tubes used. Thus, care must be taken to use the same, or similar, rotors and centrifugation conditions mentioned in this protocol while preparing the synaptosomes. Pre cool all the rotors (SS-34, Ti-45, Ti-75 and SW-28) used to prepare the synaptosome samples to 4 ºC.

Zeiss cryo-laser scanning confocal microscope (cryo-LSM 980) and Linkam cryo-stage
Turn on the HXP lamp 15-30 min prior to use for the fluorescent lamps to reach full brightness. Simultaneously, turn on the component, the PC connected to the microscope and launch the ZEN software.
To connect the Linkam stage to the microscope, launch the MTB configuration and set "Linkam" as the active configuration. Place the desired cassette transporter (single or multi-port) in the cassette prior to cooling down the cryo-stage. Connect the Linkam cryo-stage to the microscope and allow liquid nitrogen from the dewar connected to the cryo-stage to flow and cool down the Linkam chamber and stage to -196 ºC. The temperature of the dewar, chamber and stage must be constantly maintained at -196 ºC while the microscope is in use.
Set up wide field (WF): Set the Track1, Track2 and Track3 as bright field, mVenus (laser: 514 nm) and mCherry (laser: 561 nm) channels, respectively.
Set up Airyscan: Set Track1 to Ch2 for the Airyscan detector and the remaining two tracks as mVenus (laser: 514 nm) and mCherry (laser: 561 nm) channels .
Set the Pinhole and master gain at 184 µm and 500-800 V, respectively.
To obtain the confocal image of a complete grid square, set the crop area to 0.6x. To obtain a higher signal-to-noise ratio, set the pixel time 4-6, bidirectional scanning, with an averaging of 2x. These parameters should be adjusted according to specific requirements.

Fluorescence-detection size-exclusion chromatography (FSEC)
Load the analyte onto a Superose 6 column (10/30, Amersham Biosciences) that has been preequilibrated with FSEC buffer. The eluent from the size exclusion chromatography (SEC) column is passed through Shimadzu fluorometer (RF-20Axs) fitted with flow cells, as described in manufacturer's instructions. The fluorometer settings are as follows: band-pass, 3 nm/3 nm; λ_{ex}: 510 nm (mVenus) and 580 (mCherry), λ_{em}: 535 nm (mVenus) and 610 (mCherry); time increment, 1 s; integration time, 1 s; and recording time, 3,000–3,600 s. During the run, the flowrate for separation was maintained at 0.5 mL/min. A detailed description of FSEC can be found in [56].

Aquilos 2 cryo-focused ion beam-scanning electron microscope (cryo-FIB-SEM)
Purge the cooling line by flowing liquid nitrogen at 190 mg/s for at least 30 min. Purge argon lines by two purging cycles. If the shuttle is on the stage inside the chamber, remove it using a transfer rod and place it in the preparation box. Purge gas injection system (GIS) at room temperature for 30-60 seconds. Fill the heat exchanger dewar with liquid nitrogen and cool down the system by inserting the heat exchanger in the dewar. The temperature of the stage and cryo shield should be below -185 ºC and -182 ºC, respectively.

SAFETY WARNINGS

Use caution and wear appropriate PPE when working with propane/ethane 50%/50% (v/v) and liquid nitrogen.

Preparation of crude synaptosomes from vGLUT1-mVenus knock-in mouse

1. Remove the whole brains of three mice (14-23 weeks old), immediately after being euthanized, and rinse the brains in ice-cold PBS buffer pH 7.4.

2. Add 10 mL of ice-cold homogenization buffer (HB) to the freshly dissected brain (net weight of three mice brain: ~1.55 gm).

3. Prior to using a glass Teflon dounce homogenizer, rinse all its parts with deionized (DI) water followed by rinsing with the HB, and allow it to cool in ice for 10-15 min.

4. Use a loose-fitted hand-held 15 mL glass Teflon dounce homogenizer to initially prepare a crude brain lysate using 4-5 strokes while maintaining the homogenizer in ice.

5. Further homogenize the brain lysate using a motor-driven dounce homogenizer for another 5-6 strokes, with each stroke followed by ~30 sec incubation of the glass tube containing the brain lysate in ice.

6. Collect the homogenate in a sterile 15 mL falcon centrifuge tube and centrifuge at 1000*g* for 10 min at 4 ºC.

7. Gently decant the supernatant (S1) to a fresh 15 mL tube. The pellet formed is very soft, hence care should be taken not to mix it with the supernatant.

8. Centrifuge the supernatant (S1) for another 20 min at 13,000*g* at 4 ºC using high-speed polycarbonate round bottom centrifuge tubes in a SS-34 fixed angle rotor to obtain the pellet containing synaptosomes.

9 Remove the supernatant and gently resuspend the pellet in 1.5-2 mL of HB. Do not vortex.

10 Add 50-100 nM of 15F1 Fab-mCherry to the resuspended synaptosome homogenate and gently nutate it for 1 h at 4 ºC.

Preparation of sucrose/Ficoll/Percoll density gradient

11 Prepare the required volume and concentration of sucrose/Ficoll/Percoll gradient buffers as described in "Reagent Setup" section.

12 Use a 5 mL pipette or a 14G dispensing needle attached to a 10 mL syringe to slowly create the sucrose or Ficoll density gradient layers in an open-top thin wall ultra-clear tube. For Percoll density gradient separation, use 2 mL each of the density gradient buffers to create the layers in a 10.4 mL polycarbonate bottle with cap assembly.

13 Start from the heaviest density to the lightest, working the way up from bottom to top. For optimal results, hold the tube at 45 º angle and maintain the flow as one drop at a time out of the pipette tip or needle, while creating a gentle stream of the buffer trickling down the walls of the tube.

14 After preparation of each layer, let the tube stand for ~2 min before layering the next gradient on top.

15 Once all the gradient layers are being formed, allow the tubes to sit in ice or 4 ºC for 10-15 min.

16 Layer the synaptosome homogenate on top of the density gradient layers and let sit for ~2 mins.

Density gradient centrifugation

17 For sucrose and Ficoll density gradient centrifugation, spin the tubes at 30,965*g* for 70 min at 4 ºC using a swinging bucket rotor (SW 27) in an Optima L-80 XP ultracentrifuge, Beckman Coulter. For Percoll density gradient centrifugation, use a fixed bucket rotor (Type 75 Ti in an Optima L-80 XP ultracentrifuge, Beckman Coulter) to centrifuge at 12,854*g* for 15 min at 4 ºC. It is important to set the acceleration and deceleration speed to 6 and 4, respectively, in order to avoid disturbing the separated fractions and gradient layers.

Retrieval of the enriched synaptosomes

18 Enriched synaptosomes form a visibly distinct layer in between 0.8 and 1.2 M sucrose, 8 and 14% Ficoll and 15 and 23% Percoll (S1 Fig.). In the cases of sucrose and Ficoll, wherein a thin-

walled tube is used, use an 18-gauge needle attached to a 10 mL syringe to access the synaptosome band by rupturing the side of the tube.

19 An alternative method to retrieve the synaptosomes prepared using the Percoll density gradient is to use the 14G dispensing needle attached to a 10 mL syringe. Gently remove the unwanted layers from the top until the synaptosome band can be accessed. In all instances, care should be taken not to disturb the gradient layers.

Transferring the synaptosomes to an isotonic buffer

20 To remove sucrose, Ficoll or Percoll after density gradient centrifugation, dilute the retrieved synaptosomes to at least five-fold in HB and centrifuge at 30, 000*g* for 15 min at 4 ºC using a 70 mL polycarbonate bottle assembly in a Type 45 Ti fixed angle rotor compatible with Optima L-80 XP ultracentrifuge, Beckman Coulter.

21 Remove the supernatant and resuspend the synaptosomes in PBS pH 7.4, or any desired buffer.

22 Add 5 mL PBS pH 7.5 to the synaptosomes and gently resuspend the pellet using a pipette without causing froth formation. Sufficient resuspension is critical for well-dispersed non-aggregated synaptosomes.

23 Immediately proceed to cryo-EM grid preparation because synaptosomes stored on ice or at 4 °C tend to aggregate.

Filtration of synaptosomes using a thermobarrel extruder

24 Prepare the synaptosomes as described in steps 1-19.

25 Resuspend the synaptosomes in 10 mL PBS pH 7.4.

26 Set up the extruder as described in manufacturer’s manual.

27 Use a glass Pasteur pipet to carefully apply 0.5-1 mL of the synaptosomes on top of the 1 µm filter paper in the extruder and continue for the entire volume.

28 Carefully collect the filtered synaptosomes in a clean 50 mL Falcon tube placed in ice.

29 Repeat the filtration for nine passes, each time using the filtered synaptosomes obtained from the preceding step.

30 Replace the filter after passing 3-5 mL of the synaptosomes to avoid clogging up the extruder.

FSEC analysis of glutamatergic synaptosomes

31 Before subjecting the retrieved glutamatergic synaptosome sample to FSEC analysis, solubilize the synaptosomes using digitonin containing solubilization buffer as described in steps 32-36.

32 Take 0.5-1 mL of synaptosomes and centrifuge at 15, 000 *g* for 10 min.

33 Remove the supernatant and resuspend the synaptosomes in 100 µL of TBS pH 8 buffer.

34 Add 100 µL of solubilization buffer (refer "Reagent set up") to the homogeneously resuspended synaptosomes.

35 Gently nutate the samples for 1 h at 4 °C.

36 Centrifuge the solubilized synaptosomes at 70,000 *g* for 40 min at 4 °C.

37 Collect the supernatant and analyze 70 µL by FSEC.

38 Monitor for the presence of vGLUT1-mVenus and AMPAR-15F1 Fab-mCherry signals using mVenus (λ_{ex}: 510 nm, λ_{em}: 535 nm) and mCherry (λ_{ex}: 580 nm, λ_{em}: 610 nm) channels, respectively.

Preparation of cryo-EM grids using a manual plunger

39 Prior to cryo-EM grid preparation, add 2 µL of 10 nm gold fiducialto 10 µL synaptosomes and mix well.

40 Plunge-freeze the synaptosomes using a manual plunge freezer. Apply 2.5 µL of synaptosomes to the glow-discharged side of the grid and blot away the excess liquid from the back of the grid using blotting paper.

41 After blotting, immediately vitrify the grid in propane/ethane 50%/50% (v/v) mixture, cooled liquid nitrogen. Carefully transfer grids to a grid box placed in liquid nitrogen.

Sample clipping

42 Mark the cryo-FIB-AutoGrids with an alcohol-resistant marker on the single embedded mark 90 º clockwise from the milling slot. This marking will facilitate the correct alignment of the grid in the cryo-FIB shuttle and later in the transmission electron microscope (TEM) cassette for cryo-ET.

43 Cool down the transfer station with liquid nitrogen and place the marked cryo-FIB-AutoGrid upside down in the grid mounting position.

44 Place the grid on top of the cryo-FIB-AutoGrid with the sample side of the grid facing downward.

45 Load a C-ring into the tip of a C-ring insertion tool using a tweezer and gently press the C-ring on a flat and firm surface to align it with the rim of the insertion tool.

46 Precool the C-ring loaded insertion tool in liquid nitrogenand gently push to the C-ring to clip the sample cryo-EM grid onto the cryo-FIB-AutoGrid while holding the C-ring insertion tool vertically.

47 Use a blunt-end tweezer to gently turn the clipped cryo-FIB AutoGrid upside down to ensure that the grid is clipped correctly.

Imaging glutamatergic synaptosomes using cryo-LSM

48 For imaging the glutamatergic synaptosomes on the cryo-EM grids, use a fluorescence microscope equipped with a cryo-stage. Here we used a Zeiss LSM 980 with Airyscan 2 coupled with a Linkam cryo-stage.

49 Carefully place the clipped cryo-EM grid in the Cassette Transport in the liquid nitrogen-filled Linkam cryo-stage.

50 Launch the ZEN software.

51 Turn on the lasers, select “Acquisition” from the main toolbar and acquire the image using the WF for the low magnification image of the grid using the 5X objective lens.

52 Change to the 10X objective lens and acquire a medium magnification image of the grid. At medium magnification, one-fourth of the grid can be imaged at one time. Acquire images of the areas which have the least number of broken squares. If imaging for cryo-FIB milling, it is important to obtain an image of the mid area of the grid, as this area is often the best accessible by the ion beam during cryo-FIB milling.

53 Change to the 100X objective lens. Precaution should be taken to first correctly focus the grid at 5X and 10X, so as to avoid the crashing of the objective lens of the microscope into the Linkam cryo-stage.

54 While using the 100X objective lens, set the Airyscan light path configuration to confocal image acquisition.

55 Acquire images at the desired areas and save images as .tif or .jpg files for later use during alignment in cryo-FIB-SEM.

Cryo-FIB milling of glutamatergic synaptosomes

56 Load the cryo-FIB-AutoGrid into the 45º pre tilited cryo-FIB-SEM shuttle. Make sure the milling slot of the cryo-FIB-Autogrid is aligned to the vertical position. Load the shuttle into the cryo-FIB-SEM chamber by using the transfer rod.

57 Using Maps software, acquire SEM tile set image of the grid with 550 µm horizontal field width (HFW) and 3072 x 2048 pixel (px) or higher to clearly visualize surface structures. Import fluorescent light microscope (FLM) image into Maps software and align to the SEM tile set image. There will be no apparently visible features corresponding to synaptosomes during SEM imaging. Hence, ensure that the FLM images are very well aligned to the SEM image by checking features such as the center of the grid, grid bars or broken carbon films for optimal location of the target.

58 Within the Maps software, pick the lamella sites close to the center of the grid squares based on the FLM image. During a session of 6-8 hrs as many as 10 lamellae can be prepared.

59 Sputter coat the surface of the grid with a conductive layer of platinum (Pt) for 10 sec at 30 mA and 10 Pa, followed by deposition of an additional Pt gas injection system (GIS) layer for another 15 secs to protect the lamella surface and prevent the curtaining effect.

Use AutoTEM 2.0 software for milling. Set up the milling parameters as shown in S1 Table.

60 Parameters such as milling current and depth correction need to be adjusted according to the ice thickness of the lamella sites. Final thickness is set to 350 nm so each lamella can be further milled down to 100-200 nm by manual milling.

A	B	C	D	E
Milling angle (º)	12			
Size (µm)	12x 5			
Final thickness (nm)	350			
Correction factor (%)	0.6			
	lamella thickness (Pattern offset) (µm)	Milling current (nA)	Depth correction (%)	Width overlap (Front and rear) (µm)
Rough milling	1	1	70	400
Medium milling	0.75	0.3	70	300
Fine milling	0.3	0.1	70	200
Finer milling	0.15	0.05	70	100
Polishing	0.025	0.03	80	N/A

S1 Table: Conditions for cryo-FIB milling of synaptosomes

61 In the preparation step of AutoTEM 2.0, perform eucentric tilting with a 10 º tilt step followed by lamella placement to register final lamella position.

62 In the milling step from rough Milling to finer milling, use stepwise mode to thin down each lamella site. Mill micro-expansion joints, also known as stress relief cuts, alongside the lamella to avoid lamella bending [58]. After starting the milling process, make sure the parameters are suitable for the sample and adjust the milling current or depth correction as required. The milling step would render a lamella with a thickness of ~600nm.

63 Further mill down the lamella to ~350 nm during the polishing step.

64 To obtain the final lamella within the thickness range of ~100-200 nm, manually polish using 10 or 30 pA milling current at 30 kV. Use rectangle pattern to visually inspect milled materials during thinning.

Imaging on cryo-FIB milled lamella

65 To ensure the presence of glutamatergic synaptosomes, subject the FIB-milled lamellae to

cryo-LSM imaging.

66 Repeat steps 48-55 for imaging.
