# Supplementary material for: Isolation, cryo-laser scanning confocal microscope imaging and cryo-FIB milling of mouse glutamatergic synaptosomes
Source: PLoS One. 2022 Aug 12;17(8):e0271799. doi: 10.1371/journal.pone.0271799 (PMC9374259; doi:10.1371/journal.pone.0271799)
Supplement: Attachment — Submitted filename: Response to Reviewers.docx [file pone.0271799.s003.docx]

Academic Editor
PLOS ONE

Journal Requirements:

When submitting your revision, we need you to address these additional requirements.

1. Please ensure that your manuscript meets PLOS ONE's style requirements, including those for file naming. The PLOS ONE style templates can be found at

<https://journals.plos.org/plosone/s/file?id=wjVg/PLOSOne_formatting_sample_main_body.pdf>  and

<https://journals.plos.org/plosone/s/file?id=ba62/PLOSOne_formatting_sample_title_authors_affiliations.pdf>

- The manuscript has been modified to meet PLOS ONE's style requirements.

2. In your Data Availability statement, you have not specified where the minimal data set underlying the results described in your manuscript can be found. PLOS defines a study's minimal data set as the underlying data used to reach the conclusions drawn in the manuscript and any additional data required to replicate the reported study findings in their entirety. All PLOS journals require that the minimal data set be made fully available. For more information about our data policy, please see <http://journals.plos.org/plosone/s/data-availability>.

Upon re-submitting your revised manuscript, please upload your study’s minimal underlying data set as either Supporting Information files or to a stable, public repository and include the relevant URLs, DOIs, or accession numbers within your revised cover letter. For a list of acceptable repositories, please see <http://journals.plos.org/plosone/s/data-availability#loc-recommended-repositories>. Any potentially identifying patient information must be fully anonymized.

- We have made our step-wise protocol available at protocol.io and can be accessed at dx.doi.org/10.17504/protocols.io.kxygxz5mkv8j/v1.

Important: If there are ethical or legal restrictions to sharing your data publicly, please explain these restrictions in detail. Please see our guidelines for more information on what we consider unacceptable restrictions to publicly sharing data: <http://journals.plos.org/plosone/s/data-availability#loc-unacceptable-data-access-restrictions>. Note that it is not acceptable for the authors to be the sole named individuals responsible for ensuring data access.

We will update your Data Availability statement to reflect the information you provide in your cover letter.

3. Thank you for stating the following in the Acknowledgments Section of your manuscript:

“This work was supported by the NIH (NINDS) grant 2R01NS038631 to E.G. and E.G. is an investigator with the Howard Hughes Medical Institute.”

We note that you have provided additional information within the Acknowledgements Section that is not currently declared in your Funding Statement. Please note that funding information should not appear in the Acknowledgments section or other areas of your manuscript. We will only publish funding information present in the Funding Statement section of the online submission form.

Please remove any funding-related text from the manuscript and let us know how you would like to update your Funding Statement. Currently, your Funding Statement reads as follows:

“EG NIH (NINDS) 2R01NS038631

EG Howard Hughes Medical Institute

The funders had and will not have a role in study design, data collection and analysis, decision to publish, or preparation of the manuscript.”

Please include your amended statements within your cover letter; we will change the online submission form on your behalf.

- The funding information has been removed from the Acknowledgement section (Page 9, lines 220-226) and the statement “This work was supported by the NIH (NINDS) grant 2R01NS038631 to E.G. and E.G. is an investigator with the Howard Hughes Medical Institute.” is provided on the cover letter.

4. Please include your full ethics statement in the ‘Methods’ section of your manuscript file. In your statement, please include the full name of the IRB or ethics committee who approved or waived your study, as well as whether or not you obtained informed written or verbal consent. If consent was waived for your study, please include this information in your statement as well.

- Ethics statement: All procedures were approved by the Oregon Health & Science University’s Institutional Animal Care and Use Committee (IACUC), protocol TR01_IP00000905, Synaptic Transmission and Mechanotransduction.

5. We note that you have stated that you will provide repository information for your data at acceptance. Should your manuscript be accepted for publication, we will hold it until you provide the relevant accession numbers or DOIs necessary to access your data. If you wish to make changes to your Data Availability statement, please describe these changes in your cover letter and we will update your Data Availability statement to reflect the information you provide.

- No changes needed to the Data Availability Statement.

6. Please include captions for your Supporting Information files at the end of your manuscript, and update any in-text citations to match accordingly. Please see our Supporting Information guidelines for more information: <http://journals.plos.org/plosone/s/supporting-information>

- The caption for the Supporting Information file has been added at the end of the manuscript (Pages 17, lines 395-406).

7. To comply with PLOS ONE submissions requirements, please provide the Protocols.io DOI in the Methods section of the manuscript using this format: “The protocol described in this peer-reviewed article is published on [protocols.io](http://protocols.io), <https://dx.doi.org/10.17504/protocols.io>[........] and is included for printing as supporting information file 1 with this article.” Please also provide the Protocols.io DOI in the “Protocol DOI” field of the submission form (via “Edit Submission”). For more information, please see our submission guidelines:  <https://journals.plos.org/plosone/s/submission-guidelines#loc-guidelines-for-specific-study-types>

- The statement has been added into the Methods section/S1 File.

[Note: HTML markup is below. Please do not edit.]

Reviewers' comments:

Reviewer's Responses to Questions

**Comments to the Author**

1. Does the manuscript report a protocol which is of utility to the research community and adds value to the published literature?

Reviewer #1: Yes

Reviewer #2: No

2. Has the protocol been described in sufficient detail?

Descriptions of methods and reagents contained in the step-by-step protocol should be reported in sufficient detail for another researcher to reproduce all experiments and analyses. The protocol should describe the appropriate controls, sample sizes and replication needed to ensure that the data are robust and reproducible.

Reviewer #1: Yes

Reviewer #2: Partly

3. Does the protocol describe a validated method?

The manuscript must demonstrate that the protocol achieves its intended purpose: either by containing appropriate validation data, or referencing at least one original research article in which the protocol was used to generate data.

Reviewer #1: No

Reviewer #2: No

4. If the manuscript contains new data, have the authors made this data fully available?

The [PLOS Data policy](http://www.plosone.org/static/policies.action#sharing) requires authors to make all data underlying the findings described in their manuscript fully available without restriction, with rare exception (please refer to the Data Availability Statement in the manuscript PDF file). The data should be provided as part of the manuscript or its supporting information, or deposited to a public repository. For example, in addition to summary statistics, the data points behind means, medians and variance measures should be available. If there are restrictions on publicly sharing data—e.g. participant privacy or use of data from a third party—those must be specified.

Reviewer #1: Yes

Reviewer #2: N/A

**5. Is the article presented in an intelligible fashion and written in standard English?**

PLOS ONE does not copyedit accepted manuscripts, so the language in submitted articles must be clear, correct, and unambiguous. Any typographical or grammatical errors should be corrected at revision, so please highlight any specific errors that need correcting in the box below.

Reviewer #1: Yes

Reviewer #2: Yes

6. Review Comments to the Author

Please use the space provided to explain your answers to the questions above. You may also include additional comments for the author, including concerns about dual publication, research ethics, or publication ethics. (Please upload your review as an attachment if it exceeds 20,000 characters)

Reviewer #1: The manuscript by Gogoi et al. described the workflow for the preparation of glutamatergic synaptosomes for cryo-electron tomography (cryo-ET) analysis. To identify the glutamatergic synaptosomes, the authors took advantage of the knock-in mouse brain in which presynaptic vGLUT1-mVenus is expressed and the GluA2 specific antibody fragment tagged with mCherry (15F1 Fab-mCherry). After density gradient centrifugation, the authors identified glutamatergic synaptosomes by cryo-confocal fluorescence microscopy, which visualizes the presynaptic vGLUT1-mVenus and postsynaptic AMPAR-15F1 Fab-mCherry-positive synaptosomes. Subsequently, cryo-FIB milling was used for rendering lamellae for cryo-ET analysis. Although cryo-ET studies are now topical and timely issues and the manuscript is clearly written, the actual cryo-ET data (i.e., cryo-ET imaging of this synaptosomes) is missing. So, the readers cannot evaluate this protocol.

Major comments
1. The authors should add the representative cryo-ET images of glutamatergic synaptosomes acquired by this method.

- We thank the reviewer for the suggestion and we have added a reconstructed tomogram of synaptosomes acquired by this method to the revised version of the manuscript (Page 8, lines 184-190 and Fig 6G).

2. The authors should cite the two key references in the cryo-ET studies of synapses and discuss them.
(1) Rubén Fernández-Busnadiego. Cryo-Electron Tomography of the Mammalian Synapse.
Methods Mol Biol. 2018;1847:217-224. doi: 10.1007/978-1-4939-8719-1_16.
(2) Chang-Lu Tao et al. Differentiation and Characterization of Excitatory and Inhibitory Synapses by Cryo-electron Tomography and Correlative Microscopy.
J Neurosci 2018 Feb 7;38(6):1493-1510.doi: 10.1523/JNEUROSCI.1548-17.2017.

- As suggested by the reviewer, both the references are discussed in the revised version of the manuscript as suggested by the reviewer (Page 4 and 5, lines 93-100).

Minor comment
The authors should spell out “FSEC” in the line 111 and “Pt GIS” in the lien 161.

- “FSEC” (Page 6, line 128) and “Pt GIS” (Page 8, line 181) has been spelled out in the revised version of the manuscript.

Reviewer #2: This brief protocol describes a synaptoneurosome preparation for cryoET studies. The data would be better suited as part of a research article, at present it is unclear whether this workflow will permit structural characterisation of synaptic receptor complexes.
Other comments
1. It would be helpful to present a workflow schematic in the main text figure, as it is currently not described with sufficient clarity.

- We thank the reviewer for the suggestion and we have incorporated a workflow schematic in the main text (Fig 1).

2. In the “Cryo-FIB milling of glutamatergic synaptosomes” section, fluorescent light microscope images were used for the guidance of FIB milling. An overlay image of FLM and SEM would be needed to demonstrate the alignment.

- Although fluorescence light images were used to guide the FIB-milling, we were unable to discern synaptosome-like features in the SEM images, simply due to the constraints of the imaging mode. Thus, we do not believe that an overlay of the FLM and SEM images would constructively augment the manuscript. What we did do, however, was to perform fluorescence imaging on the FIB-milled lamella, to confirm that our FLM guided milling was successful, as evidenced by the presence of adjacent or overlapping red and green fluorescence (Fig 6).

3. In main text line 138, the authors mentioned filtering of synaptosomes by using a thermobarrel extruder, was this sample used for the following EM grids preparation and FIB milling? Please clarify

- In the revised version of the manuscript, we have mentioned that the filtered synaptosomes were used for the preparation of EM grids (Page 7, lines 163 and 164)

4. a TEM image of a representative lamella and an its overlay with the corresponding light microscope image should be added to figure 5.

- TEM images were acquired on the lamella for which overlapping green and red fluorescence signal were detected in the cryo-LSM. However, we have not employed any tools or other ways to overlay the images.

5. The authors state that by following their procedure the final lamella can reach 100-200 nm thickness, a section of a reconstructed tomogram or other evidence should be added to support this statement.

- The thickness of the lamella was measured during cryo FIB milling using the AutoTEM 2.0 software and monitored using SEM. To further support the preparation of successful synaptosome lamellae, we have added a reconstructed tomogram to the revised version of the manuscript (Page 8, lines 167-173 and Fig 6G).

6. In S1 Fig, the authors mention the existence of other components in density gradient fractions ( “myelin & membranes and extrasynaptosomal mitochondria”). Western blot or other experimental evidence is needed to support the identification of those components.

- In this study, the steps for density gradient separation of synaptosomes from other components has been performed and similar results were reproduced as described in previous studies (Gray and Whittaker, 1962; Cotman and Matthews, 1971; Dunkley et al., 2008). All these studies have been cited appropriately (S1 Fig legend). Given the extensive prior documentation, we respectively assert that further analysis is not a productive use of time or resources.

7. PLOS authors have the option to publish the peer review history of their article ([what does this mean?](https://journals.plos.org/plosone/s/editorial-and-peer-review-process#loc-peer-review-history)). If published, this will include your full peer review and any attached files.

If you choose “no”, your identity will remain anonymous but your review may still be made public.

**Do you want your identity to be public for this peer review?** For information about this choice, including consent withdrawal, please see our [Privacy Policy](https://www.plos.org/privacy-policy).

Reviewer #1: No

Reviewer #2: No

[NOTE: If reviewer comments were submitted as an attachment file, they will be attached to this email and accessible via the submission site. Please log into your account, locate the manuscript record, and check for the action link "View Attachments". If this link does not appear, there are no attachment files.]

While revising your submission, please upload your figure files to the Preflight Analysis and Conversion Engine (PACE) digital diagnostic tool, <https://pacev2.apexcovantage.com/>. PACE helps ensure that figures meet PLOS requirements. To use PACE, you must first register as a user. Registration is free. Then, login and navigate to the UPLOAD tab, where you will find detailed instructions on how to use the tool. If you encounter any issues or have any questions when using PACE, please email PLOS at [figures@plos.org](mailto:figures@plos.org). Please note that Supporting Information files do not need this step.
